# An overview of systematic reviews on predictors of smoking cessation among young people

**DOI:** 10.1371/journal.pone.0299728

**Published:** 2024-03-11

**Authors:** Anasua Kundu, Nahid Sultana, Daniel Felsky, Theo J. Moraes, Peter Selby, Michael Chaiton

**Affiliations:** 1 Institute of Medical Science, University of Toronto, Toronto, Canada; 2 Centre for Addiction and Mental Health, Toronto, Canada; 3 Dalla Lana School of Public Health, University of Toronto, Toronto, Canada; 4 The Hospital for Sick Children (SickKids), Toronto, Canada; 5 Department of Paediatrics, University of Toronto, Toronto, Canada; 6 Department of Family and Community Medicine, University of Toronto, Toronto, Canada; Thamar University: Dhamar University, YEMEN

## Abstract

Understanding the factors that influence smoking cessation among young people is crucial for planning targeted cessation approaches. The objective of this review was to comprehensively summarize evidence for predictors of different smoking cessation related behaviors among young people from currently available systematic reviews. We searched six databases and reference lists of the included articles for studies published up to October 20, 2023. All systematic reviews summarizing predictors of intention to quit smoking, quit attempts, or smoking abstinence among people aged 10–35 years were included. We excluded reviews on effectiveness of smoking cessation intervention; smoking prevention and other smoking behaviors; cessation of other tobacco products use, dual use, and polysubstance use. We categorized the identified predictors into 5 different categories for 3 overlapping age groups. JBI critical appraisal tool and GRADE-CERqual approach were used for quality and certainty assessment respectively. A total of 11 systematic reviews were included in this study; all summarized predictors of smoking abstinence/quit attempts and two also identified predictors of intention to quit smoking. Seven reviews had satisfactory critical appraisal score and there was minimal overlapping between the reviews. We found 4 ‘possible’ predictors of intention to quit smoking and 119 predictors of smoking abstinence/quit attempts. Most of these 119 predictors were applicable for ~10–29 years age group. We had moderate confidence on the ‘probable’, ‘possible’, ‘insufficient evidence’, and ‘inconsistent direction’ predictors and low confidence on the ‘probably unrelated’ factors. The ‘probable’ predictors include a wide variety of socio-demographic factors, nicotine dependence, mental health, attitudes, behavioral and psychological factors, peer and family related factors, and jurisdictional policies. These predictors can guide improvement of existing smoking cessation interventions or planning of new targeted intervention programs. Other predictors as well as predictors of intention to quit smoking need to be further investigated among adolescents and young adults separately.

## Introduction

Worldwide approximately 8 million deaths and 200 million disability-adjusted life-years were attributed to smoking tobacco in the year 2019 [1]. Although smoking prevalence has decreased considerably over past years, still 1.1% of the Canadians aged 12 to 17 years and 11.4% aged 18 to 34 years reported currently smoking cigarettes in 2021 [2]. Almost 90% of the adult cigarette smokers start smoking cigarettes by the age of 18 years [3]. The risk of smoking-related disease development as well as the likelihood of dying from cancer increases as a function of duration of smoking [3,4]. However, quitting smoking at younger ages reduces this risk significantly and quitting by the age of 34 years avoids approximately 100% of the cancer mortality risk associated with continued smoking [4]. While many young people desire to quit smoking and the highest rate of quit attempts is seen among 18–24 years age group [5], most of them have a great deal of difficulty in successfully quitting smoking [5–7]. A previous longitudinal study reported that smokers may take on average 30 or more quit attempts before finally being successful in quitting [7]. Hence, it is important to explore the individual and environmental predictors that influence different smoking cessation related behaviors among populations before tobacco related morbidity and mortality are manifested.

Although the reasons and motivations to quit smoking are not much different between youths and adults (e.g., health concerns and costs) [8,9], they differ in other aspects. For example, youths are more likely to report concomitant nicotine and cannabis use, have higher nicotine and alcohol dependence, better physical functioning, and lower psychological functioning than older adults [10]. Moreover, smoking cessation is a behaviorally different process for youths in comparison to adults due to their high sensitivity to unique intrapersonal and interpersonal factors as well as different social determinants of health [3,11]. While previous systematic reviews have addressed different types of predictors of smoking cessation among youth, most of them are heterogenous with some focused on a single predictor, some included only a specific population group, and reviews conducted in different time periods over the years [12–14]. Moreover, it is not clear which predictors should be prioritized in terms of significance, and which predictors are needed to be studied further. Hence, we conducted a comprehensive overview where we included published systematic reviews on predictors of smoking cessation among the young population. It will improve our existing knowledge on this topic; guide us to identify higher-risk population; and eventually minimize smoking related mortality and morbidity by intervening in these individuals as young as possible [4]. The objective of this overview was to summarize individual and environmental factors that predict smoking cessation related behaviors among young people aged 10–35 years from currently available systematic reviews and categorize them according to their reported statistical significance.

## Materials and methods

We followed the Preferred Reporting Items for Systematic Reviews and Meta-Analyses (PRISMA) 2020 guidelines by adhering to the four-phase flow diagram and 27-item checklist for this study [15]. This protocol was registered on the Open Science Framework (https://osf.io/48mja/) [16]. The review was conducted in five steps: (1) database search and exclusion of duplicate articles, (2) review of the titles and abstracts to exclude clearly irrelevant articles, (3) in-depth full-text review of articles to determine inclusion, (4) extraction of relevant data from the included articles, and 5) synthesis of the extracted data, critical appraisal of the included articles, and certainty assessment of the evidence.

### Information sources and search strategy

We searched MEDLINE, PubMed, PsycINFO, CINAHL Plus and Scopus as sources of academic databases and Google Scholar as a source of grey literature. We conducted our initial literature search in September 26, 2022, but later updated our search in October 20, 2023 by using various combinations of medical subject headings (MeSH terms) and keywords (e.g., “smoking cessation”, “cigarette*”, “quit”, “abstinence”, “predict*”, “risk factors”, “correlate*”, “associate*”, “emerging adulthood”, “youth*”, “adolescent*”, and “young adult*”) (see S1 Appendix). From the initial Google Scholar search, first 200 results were considered for title and abstract screening, because we did not get any relevant article following this threshold. Later, we additionally screened first 100 results of the most recently published articles from the updated Google Scholar search. All search results were further limited to review articles on humans and English language papers. We also searched the reference lists of the included articles. One reviewer (AK) conducted both academic and grey literature search and imported the articles to the Covidence workflow platform where duplicate articles were automatically removed. Another reviewer (NS) searched the reference lists of the finally included articles looking for any potential paper which matched the eligibility criteria.

### Eligibility criteria

We included any systematic reviews which summarized factors possibly influencing smoking cessation related behaviors including intention to quit smoking, quit attempts and smoking abstinence among young people between age 10 and 35 years. Hence, other types of literature reviews including scoping and narrative reviews were excluded. Although World Health Organization (WHO) has defined people between age 10 and 24 years as adolescents and young adults [17], several studies identified people up to age 35 years as young adults [18–20]. Moreover, previous research has shown that quitting smoking by 34 years significantly lowers smoking mortality and morbidities [4]. Hence, we decided to expand our target population to people between the ages of 10 and 35 years, so that the findings of this study could be applicable for a broader group of young population. We also included reviews where the target population included people between 10 and 35 years as sub-population, or the review assessed predictors of smoking cessation as one of the various other outcomes. We excluded systematic reviews on 1) efficacy of smoking cessation interventions; 2) predictors of adverse health effects of smoking; 3) predictors of smoking prevention and other smoking behaviors such as smoking initiation, escalation, and smoking prevalence; 4) predictors of other tobacco products (OTPs) cessation; and 5) predictors of cessation of dual use of cigarettes and e-cigarettes or polysubstance use. We excluded reviews that assessed the efficacy of smoking cessation interventions because most of the studies included in these types of reviews were randomized controlled trials [21,22], which are most suitable for examining cause and effect relationship. However, rather than identifying which intervention will be more effective for causing smoking cessation, we intended to summarize the individual and environmental risk factors that influence young peoples’ smoking cessation related behaviors.

### Selection process

Two reviewers (AK and NS) independently screened each title and abstract in accordance with the inclusion and exclusion criteria. Next, each full text of the remaining articles was reviewed by the two reviewers (AK and NS) and any disagreements were resolved by further discussions among them.

### Data collection process, data items, and effect measures

We used a custom-made data extraction form to extract relevant data on general characteristics of the included reviews (author and year, number of databases searched, search period, number of included studies, study design of included studies, age group, primary outcome, number of predictors identified, key findings, special features and critical appraisal score). For any given review, we also extracted data on all predictors (both statistically significant and insignificant) of smoking cessation related behaviors reported in the review. The included reviews reported statistical significance of the predictors using either a narrative data synthesis approach, or by considering p-values < 0.05 or 95% confidence intervals reported in their included studies. We also extracted data on the direction of association of the predictors as reported in the reviews, which were either positive or negative or inconsistent. One particular review [12] did not report the direction of association of their identified predictors clearly. We contacted the corresponding author enquiring about details of the findings but did not receive any responses. Hence, we documented the direction of association for these predictors as unclear. For the reviews which assessed predictors of different cigarette or tobacco use related behaviors, we limited our data extraction to the studies included in their review that evaluated only smoking cessation behaviors. While one reviewer (AK) extracted the data from all final included reviews, another reviewer (NS) checked for accuracy of the extracted data. Disagreements were resolved by discussion between the reviewers.

### Synthesis methods

We grouped outcomes in the included reviews into two categories: intention to quit smoking and smoking abstinence/quit attempts. While intention to quit smoking was indicated by future intention to quit among current smokers, smoking abstinence was usually defined by the reviews as successfully quitting smoking for a defined time-period such as 6 months. Some reviews used the term ‘smoking cessation’ as their outcomes which was non-specific and could be interpreted as either smoking abstinence or previous quit attempts. So, we combined these outcomes with smoking abstinence and made a category named smoking abstinence/quit attempts. Most of the included reviews used broader age groups as target population. Hence, we made three categories of overlapping age groups and summarized the predictors of smoking abstinence/quit attempts for individual age groups.

By following the categorization method used in a previous systematic review [14], each identified predictor was further categorized into the following groups based on the frequency of their reported significant association in the reviews: 1) probable predictor (the predictor was reported by at least 2 reviews and found statistically significant in at least 50% of the reviews); 2) possible predictor (statistical significance was observed in only one review or <50% of the total reviews); 3) insufficient evidence (when the predictor was reported by only one review and no statistically significant association was found); 4) probably unrelated (no statistically significant effect identified in at least 2 reviews). We determined the direction of effect for each individual predictor as positive or negative based on the reported direction of association observed in majority of the reviews. For any given predictor, if an unclear association was identified for one particular review [12], we ignored it and considered the direction of association found in other reviews while determining the final direction of effect. If a predictor with unclear direction of association was examined by a single review, we grouped it as ‘insufficient evidence’ factor. An additional category- ‘inconsistent direction’ was added to the previous 4 categories to represent the predictors for which 1) equal number of reviews reported positive and negative associations; or 2) equal number of reviews reported positive/negative and inconsistent associations. Data synthesis was conducted by one reviewer (AK) and checked for accuracy by another reviewer (NS).

### Quality assessment

For quality assessment of the included reviews, we used the Joanna Briggs Institute (JBI) critical appraisal checklist for systematic reviews [23]. We choose the JBI critical appraisal checklist instead of other critical appraisal tools (e.g., A measurement tool to assess systematic reviews (AMSTAR) [24] or Risk of bias in systematic review (ROBIS) [25]), because the applicability of this tool is wide and more appropriate for systematic review of non-experimental studies [26,27]. It contains an 11-item checklist, where for each item appraised, we assigned a score of 1, if the criterion was met, and 0, if the criterion was not met or was unclear. We modified the 11-item checklist to a 10-item checklist for selective reviews of qualitative studies, because item no. 9 was not applicable for these type of reviews [23]. Finally, all critical appraisal scores were reported as a percentage of the assigned total numerical scores instead of individual points. We followed a recent overview [28] to present our results for the JBI critical appraisal checklist and determined an overall score of at least 70% indicating satisfactory methodological quality. Two reviewers (AK and NS) independently scored all reviews, and any disagreement was resolved by discussion.

### Certainty assessment and overlapping between the reviews

As the included reviews did not conduct any meta-analysis and mostly presented their findings by narrative data synthesis, we used the Confidence in Evidence from Reviews of Qualitative Research (GRADE-CERQual) approach [29] for assessing certainty or confidence in the body of evidence. Although the GRADE-CERQual [29] is mainly built for conducting systematic review of primary studies, we followed similar approach applied in previous research [30] and recommendations on adapting existing tools for assessing certainty of the evidence in an overview [31]. The certainty assessment in GRADE-CERQual is based on four components- methodological limitations, coherence, adequacy of data, and relevance [29]. We evaluated the confidence in evidence on categories of the predictors for each individual outcome by assessing whether the involved reviews had any concerns regarding these four components. Based on the judgements made for each of the four components, an overall assessment of certainty or confidence was made as high, moderate, low, or very low confidence [29]. Two reviewers (AK and NS) independently provided their judgements, and any disagreement was resolved by discussion.

As the finally included reviews addressed the same research question (e.g., population, exposure, and outcomes) and search period of these reviews overlapped with each other, we anticipated that some primary studies were included in multiple reviews, which might introduce bias in the results [32]. Hence, we measured the degree of overlapping between the reviews by using the corrected covered area (CCA) method as directed in the Pieper et al., 2014 [32]. Covered area (CA) and CCA are calculated by:

$$CA=\frac{N}{rc}$$

$$CCA=\frac{N-r}{rc-r}$$

Here, *N* is the number of total studies across all reviews (including multiple counting of the same study), *r* is the number of primary studies and *c* is the number of reviews. The CCA value 0–5 indicates slight overlap, 6–10 moderate overlap, 11–15 high overlap and >15 means very high overlap between the reviews [32].

Although one of our included reviews [12] reported including total 51 studies in the final review, we could only retrieve 18 studies from their reference list. Our efforts to get the full reference list failed to get any responses from the authors of the review [12]. Hence, we counted only the retrieved 18 original studies for measuring the final CA and CCA measures.

## Results

### Study selection

**Fig 1:**
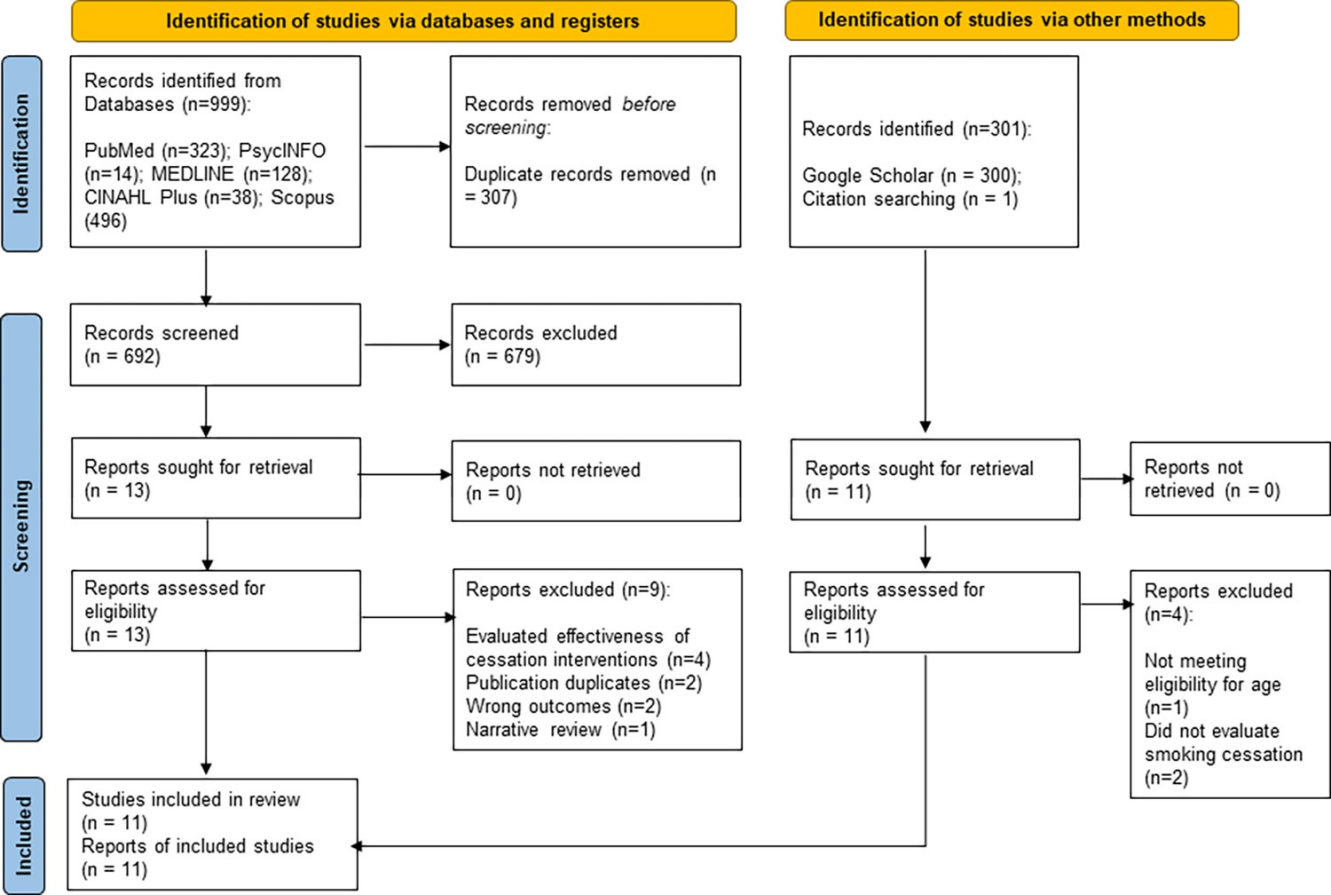
PRISMA flow diagram demonstrating selection of studies.

The search of academic electronic databases yielded 999 articles. Following removal of 307 duplicates, a total of 692 articles were reviewed by title and abstract screening. An additional 300 articles were screened through Google Scholar search and 1 article was added from searching reference lists of the final included articles. Finally, a total of 24 articles were selected for full-text screening. After removing 13 articles for various reasons (Fig 1), we included total 11 articles [11–14,33–39] in the final review. The detailed selection process of the articles is presented in the PRISMA flow diagram (Fig 1).

### Study characteristics and results of individual studies

The summary statistics of the included studies and general characteristics of individual reviews are presented in Tables 1 and 2 respectively. Out of 11 final systematic reviews [11–14,33–39], only 2 [12,33] were published before 2010, the remaining 9 were published between 2011 and 2022. Only 2 reviews [35,37] assessed predictors of intention to quit smoking, while all reviews [11–14,33–39] identified predictors of smoking abstinence/quit attempts. Four [13,34,36,37] out of 11 reviews included only 1–5 studies on smoking cessation in their review, 2 reviews [35,39] included 6–10 studies, 2 reviews [11,33] included 11–20 studies, and 3 reviews [12,14,38] included >20 studies. The highest number of studies included in one review [12] was 51 studies, however, only 18 of them were retrievable. The second highest number of studies included in one review [38] was 39. Five reviews [13,34–37] detected <20 predictors, while 4 reviews [11,12,33,38] detected 21–50 predictors and 2 reviews [14,39] detected >51 predictors. The highest number of predictors identified by a single review [14] was 67 (Table 2).

**Table 1:** Summary statistics of included reviews (N = 11).

Characteristics	Number of reviews (n)
**Outcome**	
Intention to quit	2
Smoking abstinence/ quit attempts	11
**Years published**	
Before 2010	2
2011–2022	9
**No of studies included**	
1–5	4
6–10	2
11–20	2
>20	3
**No. of predictors identified**	
<20	5
21–50	4
>51	2
**Total critical appraisal score**	
<50%	1
50%-70%	3
>70%	7
**Mean (standard deviation) of % of critical appraisal score**	71.4 (16)
**95% confidence interval of critical appraisal score**	60.6–82.1
**Overlapping between reviews**	
% of overlap	0.05
Covered area (CA)	0.10
Corrected covered area (CCA)	0.01
Interpretation of overlap	Slight

**Table 2:** General characteristics of all included reviews (N = 11).

Author and year	No. of databases searched	Search period	No. of included studies	Age group (years)	Primary Outcome	No. of predictors identified	Key findings	Special features	Critical appraisal score (%)
**Study design of included studies: Prospective longitudinal studies**									
Vallata et al., 2021 [14]	2	2010–2018	34	10–24	Smoking abstinence	67	Multiple individual and environmental factors were identified and divided into various categories, most common predictor identified was age.	Updated review of Cengelli et al., 2012, broadened definition of smoking abstinence.	55
Cengelli et al., 2012 [39]	2	1984–2010	9	10–29	At least 6-months Smoking abstinence	55	Multiple individual and environmental factors were identified and divided into various categories	-	55
Sussman et al., 2003 [33]	2	1970–2001	17	12–28	Self-initiated smoking cessation/quit attempts (non-specific)	25	Multiple individual and environmental factors were identified.	-	64
**Study design of included studies: Mixed (Longitudinal, cross-sectional, and qualitative studies)**									
Bader et al., 2007 [12]	Not Reported	1990–2006	51	18–24	Smoking cessation/quit attempts (non-specific)	25	Multiple individual and environmental factors were identified.	For most of the predictors, did not provide direction of association; only 18 out of 51 papers were retrievable from the study.	45
Hana et al., 2018 [11]	2	1999–2018	11	12–21	Smoking abstinence	24	Multiple interpersonal factors were identified.	One of the final 11 studies was a systematic review (Cengelli et al., 2012).	81
Notley et al., 2022 [13]	8	2004–2020	2	<18	Smoking cessation/quit attempts (non-specific)	1	Flavoured e-liquid had inconsistent association with quitting smoking.	-	91
**Study design of included studies: Cross-sectional studies**									
Kjeld et al., 2021 [36]	3	2011–2021	1	<30	Smoking cessation/quit attempts (non-specific)	2	Tobacco price increase are associated with increased quitting, males and females are similarly affected.	Population was from high-income countries.	72
Huang et al., 2017 [37]	4	Up to 2016	4	12–18	Intention to quit smoking, Smoking cessation/quit attempts (non-specific)	2	Flavoured tobacco product use was associated with lower intent to quit smoking	Among the final 40 articles, only 4 was on intention to quit smoking among young people.	81
**Study design of included studies: Qualitative studies**									
Twyman et al., 2014 [34]	4	Up to 2014	1	<21	Smoking cessation/ quit attempts (non-specific)	5	Living in disadvantaged community along with several other environmental factors are barriers to smoking cessation	Population was youth at risk or youth living in disadvantaged community, examined only the barriers of smoking cessation	80
Bitar et al., 2023 [38]	6	2000–2020	39	10–24	Smoking cessation/quit attempts	39	Multiple individual and environmental factors were identified.	-	90
Tombor et al., 2015 [35]	4	Up to 2013	10	16–34	Intention to quit, Long-term abstinence	4	Smoker identities influences intention to quit and abstaining from smoking, the direction of association is related to the positive or negative feelings regarding smoker identity	-	80

### Synthesis of results on predictors of smoking cessation

We identified a total of 123 predictors, of which 4 were predictors of intention to quit smoking and 119 were predictors of smoking abstinence/quit attempts. There was no ‘probable’ predictor for intention to quit smoking, because the low number of studies (n = 2) examining this outcome focused on completely different factors. Hence, it did not meet our criteria of ‘probable’ predictors and we categorized these 4 factors as ‘possible’ predictors. Among them, self-recognizing smoker identity, and interactions between smoker identity and other identity like being a mother increased the likelihood, while positive risk acceptance with smoker identity decreased the likelihood of having intention to quit smoking among 16–34 years age people [35]. Additionally, using flavored tobacco was found to lower the probability of future intention to quit smoking among 12–18 years age group [37].

Tables 3–5 and S2 Appendix presents the distribution of 119 predictors of smoking abstinence/quit attempts by 3 overlapping age groups- ~12–21 years, ~16–34 years, and ~10–29 years. While the predictors for the first two age groups were very small in number, the four reviews [14,33,38,39] conducted on the ~10–29 years age group identified on an average of 46.5 predictors. No ‘probable’ and ‘probably unrelated’ predictors were found for ~12–21 years age group, while 22 ‘possible’ predictors, 3 predictors of ‘insufficient evidence’, and 1 predictor of ‘inconsistent direction’ were identified. Among the predictors identified for the ~16–34 years age group, 2 were ‘probable’, 6 were ‘possible’, 17 had ‘insufficient evidence’, and 2 were ‘inconsistent direction’ predictors. The 2 ‘probable” predictors were self-efficacy/confidence in quitting and tobacco price increase, both were found to have positive direction of association with smoking abstinence/quit attempts (Table 3). No ‘probably unrelated’ predictors were found for this age group.

**Table 3:** ‘Probable’ predictors of smoking abstinence/quit attempts by different age groups.

Age groups*	Predictors with positive association	Predictors with negative association
~12–21 years	-	-
~16–34 years	Self-efficacy/confidence in quitting, Tobacco price increase.	-
~10–29 years	Older age at smoking initiation, Higher education, Married/living with partners, Pregnancy/becoming parent, Self-efficacy/confidence in quitting, Good self-management skills, Good self-perceived general health, Parental monitoring, Parental support, Parents/family members quitting, Friends/peers quitting, Peer support, Tobacco price increase, Restricting cigarette availability, Ban on cigarette coupons.	Level of nicotine dependence, Cravings, Frequency of smoking, Intensity of smoking, Susceptibility to smoking, Pro-smoking attitudes, Previous history of cigarette use, Alcohol use, Other substance use, Stress level, Depression, Family/household smoking, Peer smoking, Social acceptability of smoking.

* Age groups had overlaps with each other.

**Table 4:** ‘Possible’ predictors of smoking abstinence/quit attempts by different age groups.

Age groups*	Predictors with positive association	Predictors with negative association
~12–21 years	Older age at smoking initiation, Self-efficacy/confidence in quitting, Anti-smoking or negative beliefs about smoking, Good cognitive coping skills.	Duration of smoking, Frequency of smoking, Nicotine dependence, Withdrawal symptoms, Cravings, Blood Nicotine level, Smoking susceptibility, Believing that quitting smoking is forever, Stress, depression, Low emotional control, Family/household smoking, Peer smoking, Social acceptability of smoking, High prevalence of smoking in the community, Lack of health and other professional support, Disadvantaged community x stress level, Disadvantaged community x smoking to control emotion.
~16–34 years	High SES, Intention to quit smoking, Cigarette price increase x sex (male/female).	Long working hours, Family/household smoking, Peer smoking.
~10–29 years	Good school performance, High socio-economic status, Single parent household, Living with parents, Household restriction of smoking, Importance of quitting, Smoking reduction, Self-concern, High harm perception of smoking, Anti-smoking or negative beliefs about smoking, Good decision-making skills, Good self-perceived mental health, Low emotional control, Regular physical activity, Social control of tobacco use, Avoiding people who smoke, Attending fewer different schools, Involvement in extracurricular activities/ keeping busy, Non-judgmental and approachable counselors, Ensuring confidentiality while taking cessation services, Prohibit smoking in premises.	Long working hours, Living with children, Lack of health and other professional support, Daily smoking, Past quit attempts, Withdrawal symptoms, CYP2A6 slow nicotine metabolism, Drug selling, Better diet, Perceived lifestyle incongruence, Believing that quitting smoking is forever, Concerned about weight gain on quitting, Boredom, Low emotional control, Seeing others smoking, Others offer to smoke, Lack of enforcement of anti-smoking laws, Period of process of change.

* Age groups had overlaps with each other.

**Table 5:** ‘Insufficient evidence’, ‘probably unrelated’ and ‘inconsistent direction’ predictors of smoking abstinence/quit attempts by different age groups*.

Predictor type	~12–21 years	~16–34 years	~10–29 years
Insufficient evidence	Recent smoking history, Alcohol use, Other substance use	Older age at smoking initiation, School performance, Frequency of smoking, Intensity of smoking, Nicotine dependence, Alcohol use, Physical activity, Self-perceived general health, Harm perception of smoking, Self-perceived mental health, Problem behavior, Rebellious/delinquent/deviant, Living with children, Pregnancy/becoming parent, Presence of smoke-free policies, Restricting cigarette availability, Cigarettes ads and sales bans on mass media	Employment, Puberty timing, Age at first daily smoking, Family history of mental issues, Family history of drug or alcohol use, Being victimized (sexual and non-sexual), Urbanicity, Expired CO level, Abstinence at least 30 days post quit date**, OTPs use, Sleep, Blood pressure, Salivary cortisol level, Tobacco symptoms, Perceived prevalence of smoking, Dysthymia, Anxiety, ADHD, Personality disorder, Criminal behavior, Problem behavior, Rebellious/delinquent/deviant. Thrill-seeking tendency, Having people helping during financial need, Community engagement, Becoming parents x sex.
Probably unrelated	-	-	Perceived reasons for quitting, School engagement.
Inconsistent direction	E-cigarette use	Education, Employment	Age, Sex, Race/ethnicity, Parental education, EC use, Cannabis use, BMI, Smoker identity, Support from others, Intention to quit smoking, Access to NRT, Presence of smoke-free legislations, Pictorial warnings on packages, Exposure to cessation campaign.

* Age groups had overlaps with each other.

** Predictor for long-term abstinence.

Abbreviation: CO, Carbon monoxide; ADHD, attention deficit hyperactivity disorder; NRT, Nicotine replacement therapy.

For the ~10–29 years age group, we detected 29 ‘probable’, 39 ‘possible’, 26 ‘insufficient evidence’, 2 ‘probably unrelated’, and 14 ‘inconsistent direction’ predictors of smoking abstinence/quit attempts. The ‘probable’ predictors included a wide variety of factors including socio-demographic factors, nicotine dependence, attitudes regarding smoking, mental health and psychological factors, behavioral factors, peer and family related factors, and policy related factors. (Table 3). The socio-demographic factors- older age at smoking initiation, higher education, married/living with partners, and pregnancy/becoming parent had positive association with smoking abstinence/quit attempts, while factors related to dependence- level of nicotine dependence, cravings, frequency, and intensity of smoking had negative association. Higher self-efficacy/self-confidence in quitting increased the likelihood, but having pro-smoking attitudes, and susceptibility to smoking reduced the likelihood of smoking abstinence/quit attempts. Among the mental health and psychological factors, good self-management skills had a positive association, and stress level and depression had negative association. Additionally, having good self-perceived general health increased the probabilities of smoking abstinence/quit attempts, while previous history of cigarette use, alcohol or other substance use lowered the likelihood of smoking abstinence/quit attempts. Among the peer and family related factors, parental monitoring, parental or peer support, and parents/family members quitting, friends/peers quitting were positive predictors, and family/household smoking, peer smoking, and social acceptability of smoking were negative predictors. Additionally, increasing tobacco prices, restricting cigarette availability, and ban on cigarette coupons were found to have positive associations with smoking abstinence/quit attempts (Table 3).

### Quality assessment findings

The average critical appraisal score of the 11 reviews was 71.4% with a standard deviation of 156%, and 95% confidence interval of 60.6% to 82.1% (Table 1). Seven reviews [11,13,34–38] had a total critical appraisal score of at least 70%, indicating satisfactory methodological quality, while two [13,38] reviews were considered to have a score of at least 90% (Table 2). Three reviews [14,33,39] had a critical appraisal score between 50 and 69% and one review [12] had a score of less than 50%. None of the reviews assessed the likelihood of publication bias in their review. Thirty percent (n = 3) of the reviews used adequate sources for searching studies and 45% (n = 5) of the reviews had an appropriate search strategy. Seventy-three percent of the reviews had conducted appropriate critical appraisals by two or more reviewers. Approximately 80% to 90% of the reviews had clear and explicit research questions, appropriate inclusion criteria, adequate methods of data extraction and data synthesis. All reviews provided recommendations for policy and/or practice based on their findings and direction for future research (see S1 Table).

### Certainty assessment and overlapping between the reviews

Tables 6 and S2 of the Supporting Information summarizes the findings and evidence profile on individual components of the GRADE-CERQual certainty assessment. Overall, we had moderate confidence on the ‘probable’, ‘possible’, ‘insufficient evidence’, and ‘inconsistent direction’ predictors of smoking abstinence/quit attempts. However, our confidence on the ‘probably unrelated’ factors of smoking abstinence/quit attempts and 4 ‘possible’ predictors of intention to quit smoking were low.

**Table 6:** GRADE-CERQual summary of findings on certainty of the evidence.

Summary of findings	Reviews contributing to the findings (citations)	CERQual assessment of confidence in the evidence	Explanation of CERQual assessment
‘Probable’ predictors of smoking abstinence/quit attempts were identified	[12,14,33,35,36,38,39]	Moderate confidence	1 review with serious methodological limitations and moderate concerns on the coherence of data, 3 reviews with moderate methodological limitations, and 3 reviews with minor methodological limitations.
‘Possible’ predictors of smoking abstinence/quit attempts were identified	[11–14,33–39]	Moderate confidence	1 review with serious methodological limitations and moderate concerns on the coherence of data, 3 reviews with moderate methodological limitations, and 7 reviews with minor methodological limitations.
‘Insufficient evidence’ factors of smoking abstinence/quit attempts were identified	[11–14,33–39]	Moderate confidence	1 review with serious methodological limitations and moderate concerns on the coherence of data, 3 reviews with moderate methodological limitations, and 7 reviews with minor methodological limitations.
‘Probably unrelated’ factors of smoking abstinence/quit attempts were identified	[14,33,38,39]	Low confidence	3 reviews with moderate methodological limitations and high concerns regarding adequacy of data.
‘Inconsistent direction’ factors of smoking abstinence/quit attempts were identified	[11,12,13,14,33–39]	Moderate confidence	1 review with serious methodological limitations and moderate concerns on the coherence of data, 3 reviews with moderate methodological limitations, and 7 reviews with minor methodological limitations.
‘Possible’ predictors of intention to quit smoking were identified	[35,37]	Low confidence	2 reviews with high concerns regarding the adequacy of data.

We identified a total of 138 primary or index publications out of the 11 reviews [11–14,33–39] (see S3 Table). We found only 7 primary studies which were included in multiple reviews. The percentage of overlap was 0.05%, CA was 0.10 and CCA value was 0.01. Hence, the degree of overlap between the reviews was slight or minimal.

## Discussion

In this overview, we identified 119 predictors of smoking abstinence/quit attempts among young people of 3 overlapping age groups between 10 and 35 years and categorized them into 5 different groups. Among them, we have moderate confidence in support of all predictors except ‘probably unrelated’ factors (Tables 3–5). However, we found only 4 ‘possible’ predictors of low confidence for intention to quit smoking. Finding out these predictors is important to plan effective smoking prevention and intervention programs targeting young smokers who have a higher probability of showing future intention to quit. While several studies have already been published on this topic [40,41], we need well-designed comprehensive systematic reviews to summarize these predictors. Additionally, the ‘possible’, ‘insufficient evidence’, and ‘inconsistent direction’ predictors of smoking abstinence/quit attempts (Table 5) should be further investigated by future longitudinal research to determine their direction of effects or statistically significant association with smoking cessation.

Our findings on the ‘probable’ predictors of smoking abstinence/quit attempts have great implications for smoking cessation programs and policies. We found that young people aged ~10–29 years and who had lower level of nicotine dependence and cravings, had a higher chance of quitting smoking (Table 3). Similarly, we also found that high frequency and intensity of smoking act as barriers for smoking abstinence/quit attempts. Hence, ‘cutting down’ on smoking or gradual cessation approach targets lowering level of nicotine dependence and increasing probability of quitting. Although a recent meta-analysis showed that the ‘cold turkey’ or abrupt cessation approach is more effective for achieving long-term abstinence than ‘cutting down’ on smoking [42], people who find it hard to quit might better engage with the later one [43]. Our findings showed that stress and depression act as barriers of quitting (Table 3). Previous research also found that smoking cessation lowers stress level and improves depression [44,45]. This feature suggests that integrating smoking cessation with the treatment of mental health conditions might be a beneficial intervention approach for young people [46]. Similarly, not using alcohol or other substances were seen to increase probability of smoking abstinence/quit attempts (Table 3). These factors should be taken into consideration while modifying personal behaviors to increase the likelihood of quitting and planning smoking cessation interventions for young polysubstance users who face unique challenges while quitting [47,48].

Additionally, we found that higher self-confidence in quitting, good self-management skills, and being less susceptible to smoking increased probabilities of smoking abstinence/quit attempts among ~10–29 years age young people. Self-efficacy/confidence in quitting also came out as a ‘probable’ predictor of ~16–34 years age group. These factors have considerable public health policy implications. Experimental studies have found evidence that programs focusing on building self-confidence and self-control, changing attitude towards smoking, and informing about related health risk of smoking were effective in increasing intention to quit and smoking cessation rate among adolescents [49,50]. Undertaking smoking cessation campaigns and incorporating these measures to minimize pro-smoking attitudes in the society might benefit at the individual, community, and national level. While peer smoking or family or household smoking are established predictors of smoking initiation [51], our findings show that they also may act as barriers for smoking abstinence/quit attempts (Table 3). Similarly, parental monitoring or support, seeing parents or families or friends quitting, as well as peer support, and low social acceptability of smoking is linked with smoking cessation among young people. This suggests that group therapy-based smoking cessation programs might work better than individual interventions for young smokers who want to quit smoking [52].

Among the other ‘probable’ predictors, starting smoking at an older age, tobacco price increase, restricting cigarette availability, and ban on cigarette coupons were seen to promote cessation in ~10–29 years age people (Table 3). While the later three factors are already been proven effective and adapted by the policy makers in several countries [53], making the minimum age to sell tobacco products 21 years instead of 18 might be a possible approach to delay smoking initiation and promote cessation among young people [54]. Among the socio-demographic predictors, attaining higher education was found to promote smoking abstinence/quit attempts. This finding suggests that having a social determinant of health approach for tobacco control is crucial for removing tobacco-related disparities [55]. Moreover, people with higher education have higher harm perception of smoking than those with low education [56]. Health concerns is one of the top reasons for quitting smoking among young people [57,58]; hence, public awareness program is recommended as a broader tobacco control policy [59]. Although our findings showed that being married/living with partners, pregnancy/becoming parent were positive ‘probable’ predictors, and ‘living with children’ was negative ‘possible’ predictors of smoking abstinence/quit attempts among ~10–29 years age young people, these might be more relevant for people who are older in this age group. Hence, further investigations into this association particularly among young adult population are warranted.

We could only identify 2 ‘probable’ predictors of ~16–34 years age group and no ‘probable’ predictors for ~12–21 years age group (Table 3). One of the reasons behind it was identifying the majority of the predictors by only four reviews which focused on a target population aged ~10 to 29. It also indicates that although our target population was people between age 10 and 35 years, the findings of this overview are more applicable for age 10–29 years. Hence, future reviews should look for predictors of smoking cessation related behaviors among adolescents (10–19 years) and young adults (20–35 years) separately.

Most of the reviews included in our study achieved a satisfactory (≥70%) total critical appraisal score. However, four [12,14,33,39] out of six reviews, which yielded the highest number of predictors, were found to have total critical appraisal scores less than 70%. Moreover, one [12] of these four reviews included 51 studies in their review, out of which only 18 were retrievable. They also did not provide any clear direction of association, which in turn considerably lowered our ability to determine the direction of effects for several smoking abstinence/cessation predictors. Hence, future systematic reviews should adhere to the established guidelines (e.g., PRISMA, Cochrane, JBI guideline for systematic reviews) [15,60,61] to improve their methodological qualities and increase confidence in their results. Finally, in the context of evidence-based impact of the patient engagement in public health research [62,63], future researchers should consider incorporating patient and public involvement to support quitting smoking among young people.

The findings of this overview should be considered with a few limitations. First of all, we did not have high confidence in our findings (Table 5); hence, the interpretation of these findings should be done with caution. We acknowledge that we considered a wide age group (10–35 years) as the target population. Although we divided this population into three overlapping age groups, still these age groups (i.e., ~10–29 years) encroached on both the adolescent and young adulthood periods. Hence, the readers should keep this fact in mind while interpreting the findings. While we used the term smoking abstinence/quit attempts as one of our outcomes, it does not indicate which predictors increase probability of remaining abstinent and which factors increase probability of relapse. Therefore, future systematic reviews should investigate smoking abstinence and quit attempts separately and identify predictors for each. We excluded non-English literatures, which might lead to missing reviews published in other languages. However, the search periods in the included reviews ranged from 1970 to 2023, capturing studies conducted in different time-periods and locations. Still, conducting another overview looking for reviews published in non-English language might complement our findings. Due to including highly heterogenous reviews of mainly observational studies, we could not conduct a meta-analysis. Moreover, our intention was to summarize the predictors of smoking cessation, rather than evaluating effect sizes or effectiveness of smoking cessation interventions.

## Conclusions

The findings of this overview have significant public health and policy implications. We identified a wide variety of ‘probable’ predictors of smoking abstinence/quit attempts among young people aged 10–35 years, which can be used for screening high risk population, improving existing smoking cessation interventions, or planning new targeted interventions programs. In addition, some of our identified predictors can be used for behavioral modification for increasing the likelihood of successful quitting. However, due to wide age range of 10–35 years and lack of high confidence in our findings, the interpretations should be done with caution. We also provided direction for future research by informing researchers about the predictors that need further testing (‘possible’, ‘insufficient evidence’ and ‘inconsistent direction’ predictors of smoking abstinence/quit attempts), the predictors that require further attention (predictors of intention to quit smoking) as well as the age groups that need to be focused.

## Supporting information

S1 Checklist PRISMA 2020 checklist. (DOCX)

S1 Appendix Database search strategies. (DOCX)

S2 Appendix Summary of predictors of smoking abstinence/quit attempts by different age groups. (DOCX)

S1 Table Quality assessment of included reviews using JBI critical appraisal tools for systematic review. (DOCX)

S2 Table GRADE-CERQual evidence profile to assess certainty or confidence in the body of evidence. (DOCX)

S3 Table Overlapping between reviews (N = 11) and CCA calculation. (DOCX)
