# Supplementary material for: An overview of systematic reviews on predictors of smoking cessation among young people
Source: PLoS One. 2024 Mar 11;19(3):e0299728. doi: 10.1371/journal.pone.0299728 (PMC10927074; doi:10.1371/journal.pone.0299728)
Supplement: S1 Appendix — (DOCX) [file pone.0299728.s002.docx]

***S1 Appendix. Database search strategies***

**PubMed Search**

Date: Oct 20, 2023

| **Searches** | **Results** |
| --- | --- |
| 1. "smoking cessation"[MeSH Terms] OR ("smoking"[All Fields] AND "cessation"[All Fields]) OR "smoking cessation"[All Fields] | 48,052 |
| 2. "Quit"[All Fields] AND ("cigarett"[All Fields] OR "cigarette s"[All Fields] OR "cigaretts"[All Fields] OR "tobacco products"[MeSH Terms] OR ("tobacco"[All Fields] AND "products"[All Fields]) OR "tobacco products"[All Fields] OR "cigarette"[All Fields] OR "cigarettes"[All Fields]) | 7,744 |
| 3. "quit"[All Fields] AND ("smoke"[MeSH Terms] OR "smoke"[All Fields] OR "smoke s"[All Fields] OR "smoked"[All Fields] OR "smokes"[All Fields] OR "smoking"[MeSH Terms] OR "smoking"[All Fields] OR "smokings"[All Fields] OR "smoking s"[All Fields]) | 16,938 |
| 4. ("smoke"[MeSH Terms] OR "smoke"[All Fields] OR "smoke s"[All Fields] OR "smoked"[All Fields] OR "smokes"[All Fields] OR "smoking"[MeSH Terms] OR "smoking"[All Fields] OR "smokings"[All Fields] OR "smoking s"[All Fields]) AND ("abstinence"[All Fields] OR "abstinences"[All Fields] OR "abstinent"[All Fields] OR "abstinents"[All Fields]) | 9,040 |
| 3. "risk factors"[All Fields] OR "predictor*"[All Fields] OR "correlate*"[All Fields] OR "associat*"[All Fields] | [7,](https://pubmed-ncbi-nlm-nih-gov.myaccess.library.utoronto.ca/?term=%22risk+factors%22%5BAll+Fields%5D+OR+%22predictor%2A%22%5BAll+Fields%5D+OR+%22correlate%2A%22%5BAll+Fields%5D+OR+%22associat%2A%22%5BAll+Fields%5D&sort=)585,637 |
| 4. ("adolescent"[MeSH Terms] OR "adolescent"[All Fields] OR "adolescence"[All Fields] OR "adolescences"[All Fields] OR "adolescents"[All Fields]) OR ("young adult"[MeSH Terms] OR "youth"[All Fields] OR "young adult"[All Fields]) | [2,8](https://pubmed-ncbi-nlm-nih-gov.myaccess.library.utoronto.ca/?term=%28%22adolescent%22%5BMeSH+Terms%5D+OR+%22adolescent%22%5BAll+Fields%5D+OR+%22adolescence%22%5BAll+Fields%5D+OR+%22adolescences%22%5BAll+Fields%5D+OR+%22adolescents%22%5BAll+Fields%5D%29+OR+%28%22young+adult%22%5BMeSH+Terms%5D+OR+%22youth%22%5BAll+Fields%5D+OR+%22young+adult%22%5BAll+Fields%5D%29&sort=)81,491 |
| 5. ((("smoking cessation"[MeSH Terms] OR ("smoking"[All Fields] AND "cessation"[All Fields]) OR "smoking cessation"[All Fields]) OR ("Quit"[All Fields] AND ("cigarett"[All Fields] OR "cigarette s"[All Fields] OR "cigaretts"[All Fields] OR "tobacco products"[MeSH Terms] OR ("tobacco"[All Fields] AND "products"[All Fields]) OR "tobacco products"[All Fields] OR "cigarette"[All Fields] OR "cigarettes"[All Fields]))) OR ("quit"[All Fields] AND ("smoke"[MeSH Terms] OR "smoke"[All Fields] OR "smoke s"[All Fields] OR "smoked"[All Fields] OR "smokes"[All Fields] OR "smoking"[MeSH Terms] OR "smoking"[All Fields] OR "smokings"[All Fields] OR "smoking s"[All Fields]))) OR (("smoke"[MeSH Terms] OR "smoke"[All Fields] OR "smoke s"[All Fields] OR "smoked"[All Fields] OR "smokes"[All Fields] OR "smoking"[MeSH Terms] OR "smoking"[All Fields] OR "smokings"[All Fields] OR "smoking s"[All Fields]) AND ("abstinence"[All Fields] OR "abstinences"[All Fields] OR "abstinent"[All Fields] OR "abstinents"[All Fields])) | 55,479 |
| 6. ((((("smoking cessation"[MeSH Terms] OR ("smoking"[All Fields] AND "cessation"[All Fields]) OR "smoking cessation"[All Fields]) OR ("Quit"[All Fields] AND ("cigarett"[All Fields] OR "cigarette s"[All Fields] OR "cigaretts"[All Fields] OR "tobacco products"[MeSH Terms] OR ("tobacco"[All Fields] AND "products"[All Fields]) OR "tobacco products"[All Fields] OR "cigarette"[All Fields] OR "cigarettes"[All Fields]))) OR ("quit"[All Fields] AND ("smoke"[MeSH Terms] OR "smoke"[All Fields] OR "smoke s"[All Fields] OR "smoked"[All Fields] OR "smokes"[All Fields] OR "smoking"[MeSH Terms] OR "smoking"[All Fields] OR "smokings"[All Fields] OR "smoking s"[All Fields]))) OR (("smoke"[MeSH Terms] OR "smoke"[All Fields] OR "smoke s"[All Fields] OR "smoked"[All Fields] OR "smokes"[All Fields] OR "smoking"[MeSH Terms] OR "smoking"[All Fields] OR "smokings"[All Fields] OR "smoking s"[All Fields]) AND ("abstinence"[All Fields] OR "abstinences"[All Fields] OR "abstinent"[All Fields] OR "abstinents"[All Fields]))) AND (("adolescent"[MeSH Terms] OR "adolescent"[All Fields] OR "adolescence"[All Fields] OR "adolescences"[All Fields] OR "adolescents"[All Fields]) OR ("young adult"[MeSH Terms] OR "youth"[All Fields] OR "young adult"[All Fields]))) AND ("risk factors"[All Fields] OR "predictor*"[All Fields] OR "correlate*"[All Fields] OR "associat*"[All Fields]) | 6,566 |
| 7. Search: #6 Filters: Review, Systematic Review, Humans, English | 323 |

**PsycINFO search**

Date: Oct 20, 2023

| **Searches** | **Results** |
| --- | --- |
| 1. exp Smoking Cessation/ | 14,985 |
| 2. ((smoking or cigarette*) adj2 (cessation or quit or abstinence)).tw. | 15,619 |
| 3. ((smoking or cigarette*) adj2 (cessation or quit or abstinence)).ti. | 5,884 |
| 4. exp risk factors/ | 103,449 |
| 5. exp prediction/ | 33,167 |
| 6. ((risk* or predict* or correlate* or associat*) adj2 factor*).tw. | 185,264 |
| 7. ((risk* or predict* or correlate* or associat*) adj2 factor*).ti. | 29,850 |
| 8. exp adolescent behavior/ | 4,416 |
| 9. exp emerging adulthood/ | 7,769 |
| 10. (youth* or adolescen* or (young adj2 adult*)).tw. | 415,188 |
| 11. (youth* or adolescen* or (young adj2 adult*)).ti. | 213,611 |
| 12. 1 or 2 or 3 | 19,252 |
| 13. 4 or 5 or 6 or 7 | 244,422 |
| 14. 8 or 9 or 10 or 11 | 417,703 |
| 15. 12 and 13 and 14 | 268 |
| 16. limit 15 to (human and english language and ("0800 literature review" or "0830 systematic review")) | 14 |

**MEDLINE search**

Date: Oct 20, 2023

| **Searches** | **Results** |
| --- | --- |
| 1. exp Smoking Cessation/ | 32,886 |
| 2. ((smoking or cigarette*) adj2 (cessation or quit or abstinence)).tw,kf. | 36,066 |
| 3. exp Risk Factors/ | 965,750 |
| 4. ((risk* or predict* or correlate* or associat*) adj2 factor*).tw,kf. | 1,042,795 |
| 5. exp Adolescent/ | 2,223,238 |
| 6. exp Young Adult/ | 1,015,516 |
| 7. (youth* or adolescen* or (young adj2 adult*)).tw,kf. | 539,551 |
| 8. 1 or 2 | 48,860 |
| 9. 3 or 4 | 1,620,045 |
| 10. 5 or 6 or 7 | 2,858,433 |
| 11. 9 and 9 and 10 | 2021 |
| 12. limit 11 to (english language and "review articles" and humans) | 128 |

**CINAHL Plus search**

Date: Oct 20, 2022

| **Searches** | **Results** |
| --- | --- |
| S1. (MH "Smoking Cessation") | 22,929 |
| S2. TI ((smoking or cigarette*) N2 (cessation or quit or abstinence)) OR AB ((smoking or cigarette*) N2 (cessation or quit or abstinence)) | 18,971 |
| S3. (MH "Prediction Models") | 4,069 |
| S4. (MH "Risk Factors") | 199,863 |
| S5. TI ((risk* N2 factor*) or predict* or correlate* or associat*) OR AB ((risk* N2 factor*) or predict* or correlate* or associat*) | 1,754,436 |
| S6. (MH "Adolescence") | 608,423 |
| S7. (MH "Young Adult") | 291,197 |
| S8. TI (youth* or adolescen* or (young N2 adult*)) OR AB (youth* or adolescen* or (young N2 adult*)) | 248,301 |
| S9. S1 OR S2 | 30,033 |
| S10. S3 OR S4 OR S5 | 1,824,451 |
| S11. S6 OR S7 OR S8 | 811,104 |
| S12. S9 AND S10 AND S11 | 2.763 |
| S13. S12 (**Limiters** - Human; Language: English) | 2,186 |
| S14. S13 (**Limiters** - Human; Publication Type: Systematic Review; Language: English) | 35 |
| S15. S13 (**Limiters** - Human; Publication Type: Review; Language: English) | 3 |
| S16. S14 OR S15 | 38 |

**Google Scholar search**

Date: September 26, 2022

Search terms: ('smoking cessation' OR ‘smoking abstinence’ OR 'quit smoking' OR 'quit cigarette') ('risk factor' OR predictor OR associated) (adolescent OR youth OR young adult)

Filters applied: Review article

Results: 22,000 (selected first 200 to screen)

Updated search on Oct 10, 2023: Additional articles n=7,100 (selected first 100 to screen)

**Scopus database search**

Date: Oct 20, 2023

Search terms: ( TITLE-ABS-KEY ( "smoking cessation"  OR  "smoking abstinence"  OR  "quit smoking"  OR  "quit cigarette" ) )  AND  ( TITLE-ABS-KEY ( "risk factor*"  OR  predict*  OR  correlate*  OR  associat* ) )  AND  ( TITLE-ABS-KEY ( youth*  OR  adolescent*  OR  "young adult*" ) )

Limits: English language, human, review

Results: 496
