# Supplementary material for: An overview of systematic reviews on predictors of smoking cessation among young people
Source: PLoS One. 2024 Mar 11;19(3):e0299728. doi: 10.1371/journal.pone.0299728 (PMC10927074; doi:10.1371/journal.pone.0299728)
Supplement: S2 Appendix — (DOCX) [file pone.0299728.s003.docx]

***S2 Appendix. Summary of predictors of smoking abstinence/quit attempts by different age groups.***

*Table 1. Distribution of predictors of smoking abstinence/quit attempts for individual age groups.*

| **Age group*** | **No. of studies** | **Studies** | **Average no. of predictors identified by a single study** | **Total no. of the predictors** | | | | |
| --- | --- | --- | --- | --- | --- | --- | --- | --- |
|  |  |  |  | **Probable** | **Possible** | **Insufficient evidence** | **Probably unrelated** | **Inconsistent direction** |
| ~12-21 years | 4 | Huang et al., 2017 [37];  Notley et al., 2022 [13];  Hana et al., 2018 [11];  Twyman et al., 2014 [34]. | 8 | 0 | 22 | 3 | 0 | 1 |
| ~16-34 years | 3 | Bader et al., 2007 [12];  Tombor et al., 2015 [35];  Kjeld et al., 2021 [36]. | 10.3 | 2 | 6 | 17** | 0 | 2 |
| ~10-29 years | 4 | Vallata et al., 2021 [14]; Cengelli et al., 2012 [39];  Sussman et al., 2003 [33];  Bitar et al., 2023 [38]. | 46.5 | 29 | 39 | 26 | 2 | 14 |

* Age groups had overlaps with each other.

**Bader et al., 2007 did not mention the direction of association for most of their predictors. We mentioned their direction as unclear and categorized them as ‘insufficient evidence’.

*Table 2. Directions of effects and groupings of predictors of smoking abstinence/quit attempts for different age groups.*

|  | **Age ~12-21 years*** | | | | **Age ~16-34 years*** | | | **Age ~10-29 years*** | | |
| --- | --- | --- | --- | --- | --- | --- | --- | --- | --- | --- |
| **Predictors** | **No. of studies with significant association/no.**  **of studies which evaluated**  **the factor** | **Overall direction of influence (no. of studies with direction of association)** | | **Group of the factor** | **No. of studies with significant association/no.**  **of studies which evaluated**  **the factor** | **Overall direction of influence (no. of studies with direction of association)** | **Group of the factor** | **No. of studies with significant association/no.**  **of studies which evaluated**  **the factor** | **Overall direction of influence (no. of studies with direction of association)** | **Group of the factor** |
| Increased/older age |  |  |  | |  |  |  | 1/2 | IC (1 IC) | Inconsistent direction |
| Male sex |  |  |  | |  |  |  | 3/3 | IC (1+, 1-, 1 UC) | Inconsistent direction |
| Race/ethnicity- White/non-Hispanic |  |  |  | |  |  |  | 3/3 | IC (1-, 1 IC, 1 UC) | Inconsistent direction |
| Puberty timing |  |  |  | |  |  |  | 0/1 | NA | Insufficient evidence |
| Parental education |  |  |  | |  |  |  | 1/1 | IC (1 IC) | Inconsistent direction |
| Older age at smoking initiation | 1/1 | P (1+) | Possible | | 1/1 | UC (1 UC) | Insufficient evidence | 2/2 | P (2+) | Probable |
| Older age at first daily smoking |  |  |  | |  |  |  | 0/1 | NA | Insufficient evidence |
| Duration of smoking | 1/1 | N (1-) | Possible | |  |  |  |  |  |  |
| Past quit attempts |  |  |  | |  |  |  | 1/1 | N (1-) | Possible |
| Recent smoking history | 0/1 | NA | Insufficient evidence | |  |  |  |  |  |  |
| Previous history of cigarette use |  |  |  | |  |  |  | 1/2 | N (1-) | Probable |
| Family history of mental issues |  |  |  | |  |  |  | 0/1 | NA | Insufficient evidence |
| Family history of drug or alcohol use |  |  |  | |  |  |  | 0/1 | NA | Insufficient evidence |
| Being victimized (sexual and non-sexual) |  |  |  | |  |  |  | 0/1 | NA | Insufficient evidence |
| CYP2A6 slow nicotine metabolism |  |  |  | |  |  |  | 1/1 | P (1+) | Possible |
| Education |  |  |  | | 1/1 | IC (1 IC) | Inconsistent direction | 2/2 | P (2+) | Probable |
| School performance |  |  |  | | 1/1 | UC (1 UC) | Insufficient evidence | 1/1 | P (1+) | Possible |
| Urbanicity |  |  |  | |  |  |  | 0/1 | NA | Insufficient evidence |
| Employment |  |  |  | | 1/1 | IC (1 IC) | Inconsistent direction | 0/1 | NA | Insufficient evidence |
| Annual more hours worked |  |  |  | | 1/1 | N (1-) | Possible | 1/2 | N (1-) | Possible |
| High SES |  |  |  | | 1/1 | P (1+) | Possible | 1/3 | P (1+) | Possible |
| Frequency of smoking | 1/1 | N (1-) | Possible | | 1/1 | UC (1 UC) | Insufficient evidence | 2/2 | N (2-) | Probable |
| Intensity of smoking |  |  |  | | 1/1 | UC (1 UC) | Insufficient evidence | 2/2 | N (2-) | Probable |
| Smoking reduction |  |  |  | |  |  |  | 1/1 | P (1+) | Possible |
| Expired CO |  |  |  | |  |  |  | 0/1 | NA | Insufficient evidence |
| Daily smoking |  |  |  | |  |  |  | 1/1 | N (1-) | Possible |
| Nicotine dependence | 1/1 | N (1-) | Possible | | 1/1 | UC (1 UC) | Insufficient evidence | 2/2 | N (2-) | Probable |
| Withdrawal symptoms | 1/1 | N (1-) | Possible | |  |  |  | 1/3 | N (1-) | Possible |
| Cravings | 1/1 | N (1-) | Possible | |  |  |  | 2/2 | N (2-) | Probable |
| Blood nicotine level | 1/1 | N (1-) | Possible | |  |  |  |  |  |  |
| Perceived reasons for quitting |  |  |  | |  |  |  | 0/2 | NA | Probably unrelated |
| Abstinence at least 30 days post quit date |  |  |  | |  |  |  | 0/1 | NA | Insufficient evidence |
| Use of e-cigarette | 2/2 | IC (1-, 1 IC) | Inconsistent direction | |  |  |  | 2/2 | IC (2 IC) | Inconsistent direction |
| OTPs use |  |  |  | |  |  |  | 0/1 | NA | Insufficient evidence |
| Alcohol use | 0/1 | NA | Insufficient evidence | | 1/1 | UC (1 UC) | Insufficient evidence | 3/3 | N (3-) | Probable |
| Cannabis use |  |  |  | |  |  |  | 2/2 | IC (1-, 1IC) | Inconsistent direction |
| Other substance use | 0/1 | NA | Insufficient evidence | |  |  |  | 2/2 | N (2-) | Probable |
| Drug selling |  |  |  | |  |  |  | 1/1 | N (1-) | Possible |
| Physical activity |  |  |  | | 1/1 | UC (1 UC) | Insufficient evidence | 1/1 | P (1+) | Possible |
| Better diet |  |  |  | |  |  |  | 1/1 | P (1+) | Possible |
| Perceived lifestyle incongruence |  |  |  | |  |  |  | 1/1 | P (1+) | Possible |
| Sleep |  |  |  | |  |  |  | 0/1 | NA | Insufficient evidence |
| Self-perceived general health |  |  |  | | 1/1 | UC (1 UC) | Insufficient evidence | 2/2 | P (2+) | Probable |
| BMI |  |  |  | |  |  |  | 1/1 | IC (1 IC) | Inconsistent direction |
| Blood pressure |  |  |  | |  |  |  | 0/1 | NA | Insufficient evidence |
| Salivary cortisol level |  |  |  | |  |  |  | 0/1 | NA | Insufficient evidence |
| Tobacco symptoms |  |  |  | |  |  |  | 0/1 | NA | Insufficient evidence |
| Involvement in extracurricular activities/keeping busy |  |  |  | |  |  |  | 1/2 | P (1+) | Possible |
| Self-efficacy/confidence in quitting /resisting smoking | 1/1 | P (1+) | Possible | | 2/2 | P (1+, 1 UC) | Probable | 4/4 | P (4+) | Probable |
| Smoking susceptibility | 1/1 | N (1-) | Possible | |  |  |  | 4/4 | N (4-) | Probable |
| Intention to quit smoking |  |  |  | | 1/1 | P (1+) | Possible | 3/3 | IC (1+, 1-, 1 IC) | Inconsistent direction |
| High harm perception of smoking |  |  |  | | 1/1 | UC (1 UC) | Insufficient evidence | 1/2 | P (1+) | Possible |
| Anti-smoking or negative beliefs about smoking | 1/1 | P (1+) | Possible | |  |  |  | 1/2 | P (1+) | Possible |
| Pro-smoking attitudes |  |  |  | |  |  |  | 2/3 | N (2-) | Probable |
| Concerned about weight gain on quitting |  |  |  | |  |  |  | 1/2 | N (1-) | Possible |
| Believing that quitting smoking is forever | 1/1 | N (1-) | Possible | |  |  |  |  |  |  |
| Importance of quitting |  |  |  | |  |  |  | 1/1 | P (1+) | Possible |
| Perceived prevalence of smoking |  |  |  | |  |  |  | 0/1 | NA | Insufficient evidence |
| Self-perceived mental health |  |  |  | | 1/1 | UC (1 UC) | Insufficient evidence | 1/1 | P (1+) | Possible |
| Stress | 1/1 | N (1-) | Possible | |  |  |  | 2/3 | N (2-) | Probable |
| Boredom |  |  |  | |  |  |  | 1/1 | N (1-) | Possible |
| Self-esteem |  |  |  | |  |  |  | 1/1 | P (1+) | Possible |
| Self-concern |  |  |  | |  |  |  | 1/1 | IC (1 IC) | Inconsistent direction |
| Depression | 1/1 | N (1-) | Possible | |  |  |  | 2/3 | N (2-) | Probable |
| Dysthymia |  |  |  | |  |  |  | 0/1 | NA | Insufficient evidence |
| Anxiety |  |  |  | |  |  |  | 0/1 | NA | Insufficient evidence |
| Low emotional control | 1/1 | N (1-) | Possible | |  |  |  |  |  |  |
| ADHD |  |  |  | |  |  |  | 0/1 | NA | Insufficient evidence |
| Personality disorder |  |  |  | |  |  |  | 0/1 | NA | Insufficient evidence |
| Problem behavior |  |  |  | | 0/1 | NA | Insufficient evidence | 0/1 | NA | Insufficient evidence |
| Criminal behavior |  |  |  | |  |  |  | 0/1 | NA | Insufficient evidence |
| Thrill seeking |  |  |  | |  |  |  | 0/1 | NA | Insufficient evidence |
| Rebellious/delinquent/deviant |  |  |  | | 0/1 | NA | Insufficient evidence | 0/1 | NA | Insufficient evidence |
| Decision making skills |  |  |  | |  |  |  | 1/1 | P (1+) | Possible |
| Self-management skills |  |  |  | |  |  |  | 1/2 | P (1+) | Probable |
| Cognitive coping skills | 1/1 | P (1+) | Possible | |  |  |  |  |  |  |
| Married/living with partners |  |  |  | |  |  |  | 3/3 | P (3+) | Probable |
| Single parent household |  |  |  | |  |  |  | 1/1 | P (1+) | Possible |
| Living with parents |  |  |  | |  |  |  | 1/1 | P (1+) | Possible |
| Living with children |  |  |  | | 1/1 | UC (1 UC) | Insufficient evidence | 1/1 | N (1-) | Possible |
| Pregnancy/becoming parent |  |  |  | | 0/1 | NA | Insufficient evidence | 1/2 | P (1+) | Probable |
| Family/sibling/Partner/household smoking | 1/1 | N (1-) | Possible | | 1/1 | N (1-) | Possible | 4/4 | N (4-) | Probable |
| Parental monitoring/control of tobacco use |  |  |  | |  |  |  | 2/2 | P (2+) | Probable |
| Household restriction of smoking |  |  |  | |  |  |  | 1/1 | P (1+) | Possible |
| Having people helping during financial need |  |  |  | |  |  |  | 0/1 | NA | Insufficient evidence |
| Parents/family members quitting |  |  |  | |  |  |  | 3/3 | P (3+) | Probable |
| Parental support |  |  |  | |  |  |  | 4/4 | P (3+, 1-) | Probable |
| Peer smoking | 1/1 | N (1-) | Possible | | 1/1 | N (1-) | Possible | 5/5 | N (5-) | Probable |
| Seeing others smoking |  |  |  | |  |  |  | 1/1 | N (1-) | Possible |
| Others offer to smoke |  |  |  | |  |  |  | 1/1 | N (1-) | Possible |
| Social acceptability of smoking | 1/1 | N (1-) | Possible | |  |  |  | 3/4 | N (3-) | Probable |
| Social control of tobacco use |  |  |  | |  |  |  | 1/1 | P (1+) | Possible |
| Friends/peers quitting |  |  |  | |  |  |  | 2/2 | P (2+) | Probable |
| Peer support |  |  |  | |  |  |  | 3/3 | P (3+) | Probable |
| Support from others |  |  |  | |  |  |  | 2/2 | IC (1+, 1 IC) | Inconsistent direction |
| Avoiding people who smoke |  |  |  | |  |  |  | 1/1 | P (1+) | Possible |
| Community engagement |  |  |  | |  |  |  | 0/1 | NA | Insufficient evidence |
| High prevalence of smoking in the community | 1/1 | N (1-) | Possible | |  |  |  |  |  |  |
| School engagement |  |  |  | |  |  |  | 0/2 | NA | Probably unrelated |
| Attending fewer different schools |  |  |  | |  |  |  | 1/1 | P (1+) | Possible |
| Presence of smoke-free policies |  |  |  | | 0/1 | NA | Insufficient evidence | 2/2 | IC (1+, 1 IC) | Inconsistent direction |
| Lack of enforcement of anti-smoking laws |  |  |  | |  |  |  | 1/1 | N (1-) | Possible |
| Prohibit smoking premises |  |  |  | |  |  |  | 1/1 | P (1+) | Possible |
| Exposure to cessation campaign |  |  |  | |  |  |  | 1/2 | IC (1 IC) | Inconsistent direction |
| Restricting cigarette availability |  |  |  | | 1/1 | UC (1 UC) | Insufficient evidence | 1/2 | P (1+) | Probable |
| Tobacco price increase |  |  |  | | 2/2 | P (2+) | Probable | 2/2 | P (2+) | Probable |
| Pictorial warnings on packages |  |  |  | |  |  |  | 1/1 | IC (1 IC) | Inconsistent direction |
| Cigarettes ads and sales bans on mass media |  |  |  | | 1/1 | UC (1 UC) | Insufficient evidence |  |  |  |
| Received cigarette coupons/offers/exemplars |  |  |  | |  |  |  | 2/2 | N (2-) | Probable |
| Period of process of change |  |  |  | |  |  |  | 1/1 | N (1-) | Possible |
| Lack of health and other professional support | 1/1 | N (1-) | Possible | |  |  |  | 1/1 | N (1-) | Possible |
| Non-judgmental and approachable counselors |  |  |  | |  |  |  | 1/1 | P (1+) | Possible |
| Ensuring confidentiality while taking cessation services |  |  |  | |  |  |  | 1/1 | P (1+) | Possible |
| Access to NRT |  |  |  | |  |  |  | 1/1 | IC (1 IC) | Inconsistent direction |
| Smoker identity x other identity (being a mother) |  |  |  | |  |  |  |  |  |  |
| Becoming parents x sex |  |  |  | |  |  |  | 0/1 | NA | Insufficient evidence |
| Disadvantaged community x stress level | 1/1 | N (1-) | Possible | |  |  |  |  |  |  |
| Disadvantaged community x smoking to control emotion | 1/1 | N (1-) | Possible | |  |  |  |  |  |  |
| Cigarette price increase x sex (male/female) |  |  |  | | 1/1 | P (1+) | Possible |  |  |  |

*Age groups had overlaps with each other.
