# Supplementary material for: An overview of systematic reviews on predictors of smoking cessation among young people
Source: PLoS One. 2024 Mar 11;19(3):e0299728. doi: 10.1371/journal.pone.0299728 (PMC10927074; doi:10.1371/journal.pone.0299728)
Supplement: S1 Table — (DOCX) [file pone.0299728.s004.docx]

***S1 Table. Quality assessment of included reviews using JBI critical appraisal tools for systematic review***

|  | **Critical appraisal tools** | **Vallata et al., 2021 [14]** | **Cengelli et al., 2012 [39]** | **Bader et al., 2007 [12]** | **Hana et al., 2018 [11]** | **Huang et al., 2017 [37]** | **Kjeld et al., 2021 [36]** | **Notley et al., 2022 [13]** | **Tombor et al., 2015 [35]** | **Twyman et al., 2014 [34]** | **Sussman et al., 2003 [33]** | **Bitar et al., 2023 [38]** | **Number and (%) of papers fulfilling the criterion** |
| --- | --- | --- | --- | --- | --- | --- | --- | --- | --- | --- | --- | --- | --- |
| 1. | Is the review question clearly and explicitly stated? | Y | Y | N | Y | Y | Y | Y | Y | Y | N | Y | 9 (82) |
| 2. | Were the inclusion criteria appropriate for the review question? | Y | Y | UC | Y | Y | Y | Y | Y | Y | Y | Y | 10 (91) |
| 3. | Was the search strategy appropriate? | N | N | UC | Y | Y | N | Y | N | Y | UC | Y | 5 (45) |
| 4. | Were the sources and resources used to search for studies adequate? | N | N | Y | N | N | N | Y | N | N | Y | N | 3 (27) |
| 5. | Were the criteria for appraising studies appropriate? | N | N | UC | Y | Y | Y | Y | Y | Y | Y | Y | 8 (73) |
| 6. | Was critical appraisal conducted by two or more reviewers independently? | N | N | Y | Y | Y | Y | Y | Y | Y | UC | Y | 8 (73) |
| 7. | Were there methods to minimize errors in data extraction? | Y | Y | Y | Y | Y | Y | Y | Y | N | UC | Y | 9 (82) |
| 8. | Were the methods used to combine studies appropriate? | Y | Y | N | Y | Y | Y | Y | Y | Y | Y | Y | 10 (91) |
| 9. | Was the likelihood of publication bias assessed? | N | N | N | N | N | N | N | NA* | NA* | N | NA* | 0 |
| 10. | Were recommendations for policy and/or practice supported by the reported data? | Y | Y | Y | Y | Y | Y | Y | Y | Y | Y | Y | 11 (100) |
| 11. | Were the specific directives for new research appropriate? | Y | Y | Y | Y | Y | Y | Y | Y | Y | Y | Y | 11 (100) |
| Total (obtained score/total score) | | 6 | 6 | 5 | 9 | 9 | 8 | 10 | 8 | 8 | 6 | 9 |  |
| Percentage (%) | | 55 | 55 | 45 | 81 | 81 | 72 | 91 | 80 | 80 | 55 | 90 |  |

Abbreviations: JBI, Joanna Briggs Institute; Y, yes; N, no; NA, not applicable; UC, unclear.

*Item not applicable for the article because of including qualitative studies in the review.
