# Supplementary material for: An overview of systematic reviews on predictors of smoking cessation among young people
Source: PLoS One. 2024 Mar 11;19(3):e0299728. doi: 10.1371/journal.pone.0299728 (PMC10927074; doi:10.1371/journal.pone.0299728)
Supplement: S2 Table — (DOCX) [file pone.0299728.s005.docx]

***S2 Table. GRADE-CERQual evidence profile to assess certainty or confidence in the body of evidence.***

| **Outcome(s)** | **Summary of findings** | **Reviews contributing to the findings** | **Methodological limitations** | **Coherence** | **Adequacy** | **Relevance** | **CERQual assessment of confidence in the evidence** | **Explanation of CERQual assessment** |
| --- | --- | --- | --- | --- | --- | --- | --- | --- |
| Smoking abstinence/quit attempts | ‘Probable’ predictors identified | Bader et al., 2007 [12];  Tombor et al., 2015 [35];  Kjeld et al., 2021 [36];  Vallata et al., 2021 [14]; Cengelli et al., 2012 [39];  Sussman et al., 2003 [33];  Bitar et al., 2023 [38]. | Moderate (One study have serious, 3 studies have moderate, 3 studies have minor limitations) | Moderate (One study have some concerns about the fit between the data from primary studies and the review finding) | No/very minor concerns | No/very minor concerns | Moderate confidence | 1 review with serious methodological limitations and moderate concerns on the coherence of data, 3 reviews with moderate methodological limitations, and 3 reviews with minor methodological limitations. |
| Smoking abstinence/quit attempts | 36 ‘possible’ predictors identified | Bader et al., 2007 [12];  Tombor et al., 2015 [35];  Kjeld et al., 2021 [36];  Vallata et al., 2021 [14]; Cengelli et al., 2012 [39];  Sussman et al., 2003 [33];  Bitar et al., 2023 [38];  Huang et al., 2017 [37];  Notley et al., 2022 [13];  Hana et al., 2018 [11];  Twyman et al., 2014 [34]. | Moderate (One study have serious, 3 studies have moderate, 7 studies have minor limitations) | Moderate (One study have some concerns about the fit between the data from primary studies and the review finding) | No/very minor concerns | No/very minor concerns | Moderate confidence | 1 review with serious methodological limitations and moderate concerns on the coherence of data, 3 reviews with moderate methodological limitations, and 7 reviews with minor methodological limitations. |
| Smoking abstinence/quit attempts | 25 ‘insufficient evidence’ factors identified | Bader et al., 2007 [12];  Tombor et al., 2015 [35];  Kjeld et al., 2021 [36];  Vallata et al., 2021 [14]; Cengelli et al., 2012 [39];  Sussman et al., 2003 [33];  Bitar et al., 2023 [38];  Huang et al., 2017 [37];  Notley et al., 2022 [13];  Hana et al., 2018 [11];  Twyman et al., 2014 [34]. | Moderate (One study have serious, 3 studies have moderate, 7 studies have minor limitations) | Moderate (One study have some concerns about the fit between the data from primary studies and the review finding) | No/very minor concerns | No/very minor concerns | Moderate confidence | 1 review with serious methodological limitations and moderate concerns on the coherence of data, 3 reviews with moderate methodological limitations, and 7 reviews with minor methodological limitations. |
| Smoking abstinence/quit attempts | 4 ‘probably unrelated’ factors identified | Vallata et al., 2021 [14]; Cengelli et al., 2012 [39];  Sussman et al., 2003 [33];  Bitar et al., 2023 [38]. | Moderate (3 studies have moderate limitations, one study have minor limitations) | No/ very minor concerns | High concerns (studies offered very thin data) | No/very minor concerns | Low  confidence | 3 reviews with moderate methodological limitations and high concerns regarding adequacy of data. |
| Smoking abstinence/quit attempts | 14 ‘inconsistent direction’ factors identified | Bader et al., 2007 [12];  Tombor et al., 2015 [35];  Kjeld et al., 2021 [36];  Vallata et al., 2021 [14]; Cengelli et al., 2012 [39];  Sussman et al., 2003 [33];  Bitar et al., 2023 [38];  Huang et al., 2017 [37];  Notley et al., 2022 [13];  Hana et al., 2018 [11];  Twyman et al., 2014 [34]. | Moderate (One study have serious, 3 studies have moderate, 7 studies have minor limitations) | Moderate (One study have some concerns about the fit between the data from primary studies and the review finding) | No/very minor concerns | No/very minor concerns | Moderate confidence | 1 review with serious methodological limitations and moderate concerns on the coherence of data, 3 reviews with moderate methodological limitations, and 7 reviews with minor methodological limitations. |
| Intention to quit smoking | 4 ‘possible’ predictors identified | Huang et al., 2017 [37];  Tombor et al., 2015 [35]. | Minor concerns (Data synthesis could be improved) | Minor concerns (some concerns about the fit between the data from primary studies and the review finding | High concerns (studies offered very thin data) | No/very minor concerns | Low confidence | 2 reviews with high concerns regarding the adequacy of data. |

Abbreviation: GRADE-CERQual, Confidence in Evidence from Reviews of Qualitative research.
