# Supplementary material for: An overview of systematic reviews on predictors of smoking cessation among young people
Source: PLoS One. 2024 Mar 11;19(3):e0299728. doi: 10.1371/journal.pone.0299728 (PMC10927074; doi:10.1371/journal.pone.0299728)
Supplement: S3 Table — (DOCX) [file pone.0299728.s006.docx]

***S3 Table. Overlapping between reviews (N=11) and CCA Calculation***

| **Serial** | **Index Publication** | **Vallata et al., 2021 [14]** | **Cengelli et al., 2012 [39]** | **Bader et al., 2007 [12]** | **Hana et al., 2018 [11]** | **Huang et al., 2017 [37]** | **Kjeld et al., 2021 [36]** | **Notley et al., 2022 [13]** | **Tombor et al., 2015 [35]** | **Twyman et al., 2014 [34]** | **Sussman et al., 2003 [33]** | **Bitar et al., 2023 [38]** |
| --- | --- | --- | --- | --- | --- | --- | --- | --- | --- | --- | --- | --- |
| **1** | Bachmann 2012 | X |  |  |  |  |  |  |  |  |  |  |
| **2** | Baskerville 2016 | X |  |  |  |  |  |  |  |  |  |  |
| **3** | Brinker 2015 | X |  |  |  |  |  |  |  |  |  |  |
| **4** | Chenoweth 2013 | X |  |  |  |  |  |  |  |  |  |  |
| **5** | Choi 2014 | X |  |  |  |  |  |  |  |  |  |  |
| **6** | Curry 2012 | X |  |  |  |  |  |  |  |  |  |  |
| **7** | Engels 2012 | X |  |  |  |  |  |  |  |  |  |  |
| **8** | Gmel 2016 | X |  |  |  |  |  |  |  |  |  |  |
| **9** | Haas 2014 | X |  |  |  |  |  |  |  |  |  |  |
| **10** | Ham 2013 | X |  |  |  |  |  |  |  |  |  |  |
| **11** | Hoie 2011 | X |  |  |  |  |  |  |  |  |  |  |
| **12** | Kennedy 2011 | X |  |  |  |  |  |  |  |  |  |  |
| **13** | Klein 2013 | X |  |  |  |  |  |  |  |  |  |  |
| **14** | Kvaavik 2014 | X |  |  |  |  |  |  |  |  |  |  |
| **15** | Lakon 2015 | X |  |  |  |  |  |  |  |  |  |  |
| **16** | Lim 2012 | X |  |  |  |  |  |  |  |  |  |  |
| **17** | Loprinzi 2014 | X |  |  |  |  |  |  |  |  |  |  |
| **18** | Loprinzi 2015a | X |  |  |  |  |  |  |  |  |  |  |
| **19** | Loprinzi 2016b | X |  |  |  |  |  |  |  |  |  |  |
| **20** | Mantey 2017 | X |  |  |  |  |  |  |  |  |  |  |
| **21** | Minary 2013 | X |  |  |  |  |  |  |  |  |  |  |
| **22** | Mukhopadhyay 2011 | X |  |  |  |  |  |  |  |  |  |  |
| **23** | Nagelhout 2013 | X |  |  |  |  |  |  |  |  |  |  |
| **24** | Schaefer 2013 | X |  |  |  |  |  |  |  |  |  |  |
| **25** | Scherphof 2013 | X |  |  |  |  |  |  |  |  |  |  |
| **26** | Schuck 2011 | X |  |  |  |  |  |  |  |  |  |  |
| **27** | Steinmetz-Wood 2017 | X |  |  |  |  |  |  |  |  |  |  |
| **28** | Walker 2014 | X |  |  |  |  |  |  |  |  |  |  |
| **29** | Wang 2017 | X |  |  |  |  |  |  |  |  |  |  |
| **30** | Wang 2018a | X |  |  |  |  |  |  |  |  |  |  |
| **31** | Wong 2010 | X |  |  |  |  |  |  |  |  |  |  |
| **32** | Wong 2011a | X |  |  |  |  |  |  |  |  |  |  |
| **33** | Wong 2016b | X |  |  |  |  |  |  |  |  |  |  |
| **34** | Yang 2015 | X |  |  |  |  |  |  |  |  |  |  |
| **35** | Tucker 2002 |  | X | X |  |  |  |  |  |  |  |  |
| **36** | Ellickson 2001 |  | X |  |  |  |  |  |  |  |  |  |
| **37** | Rhode 2004 |  | X |  |  |  |  |  |  |  |  |  |
| **38** | Ellickson 2001a |  | X | X |  |  |  |  |  |  | X |  |
| **39** | Tucker 2005 |  | X | X | X |  |  |  |  |  |  |  |
| **40** | Bricker 2009 |  | X |  |  |  |  |  |  |  |  |  |
| **41** | Mittelmark 1988 |  | X |  |  |  |  |  |  |  |  |  |
| **42** | Hansen 1985 |  | X |  |  |  |  |  |  |  | X |  |
| **43** | Chang 2006 |  | X |  |  |  |  |  |  |  |  |  |
| **44** | Breslau 1996 |  |  | X |  |  |  |  |  |  |  |  |
| **45** | Farrelly 1999 |  |  | X |  |  |  |  |  |  |  |  |
| **46** | Gilpin 2005 |  |  | X |  |  |  |  |  |  |  |  |
| **47** | Paavola 2001 |  |  | X |  |  |  |  |  |  | X |  |
| **48** | Rose 1996 |  |  | X |  |  |  |  |  |  |  |  |
| **49** | Chen 2001 |  |  | X |  |  |  |  |  |  | X |  |
| **50** | Winefield 1992 |  |  | X |  |  |  |  |  |  |  |  |
| **51** | Chassin 2000 |  |  | X |  |  |  |  |  |  |  |  |
| **52** | Ho 1998 |  |  | X |  |  |  |  |  |  |  |  |
| **53** | Chassin 1996a |  |  | X |  |  |  |  |  |  |  |  |
| **54** | Chaloupka 1996 |  |  | X |  |  |  |  |  |  |  |  |
| **55** | Czart 2001 |  |  | X |  |  |  |  |  |  |  |  |
| **56** | Tauras 2004 |  |  | X |  |  |  |  |  |  |  |  |
| **57** | Sheu 2004 |  |  | X |  |  |  |  |  |  |  |  |
| **58** | Murphy-Hoefer 2004 |  |  | X |  |  |  |  |  |  |  |  |
| **59** | Albrecht 1999 |  |  |  | X |  |  |  |  |  |  |  |
| **60** | Myers 2011 |  |  |  | X |  |  |  |  |  |  |  |
| **61** | Horn 2003 |  |  |  | X |  |  |  |  |  |  |  |
| **62** | Suqueira 2001 |  |  |  | X |  |  |  |  |  |  |  |
| **63** | Bailey 2009 |  |  |  | X |  |  |  |  |  |  |  |
| **64** | Killen 2001 |  |  |  | X |  |  |  |  |  |  |  |
| **65** | Cengelli 2012 |  |  |  | X |  |  |  |  |  |  |  |
| **66** | Brook 2010 |  |  |  | X |  |  |  |  |  |  |  |
| **67** | Windle 2001 |  |  |  | X |  |  |  |  |  |  |  |
| **68** | Falkin 2007 |  |  |  | X |  |  |  |  |  |  |  |
| **69** | Frasalinos 2013 |  |  |  |  | X |  |  |  |  |  |  |
| **70** | Tackett 2015 |  |  |  |  | X |  |  |  |  |  |  |
| **71** | Kaleta 2014 |  |  |  |  | X |  |  |  |  |  |  |
| **72** | King 2014 |  |  |  |  | X |  |  |  |  |  |  |
| **73** | Kostova 2012 |  |  |  |  |  | X |  |  |  |  |  |
| **74** | Evans-Polce 2018 |  |  |  |  |  |  | X |  |  |  |  |
| **75** | Chen 2019 |  |  |  |  |  |  | X |  |  |  |  |
| **76** | Amos 2005 |  |  |  |  |  |  |  | X |  |  | X |
| **77** | Berg 2010 |  |  |  |  |  |  |  | X |  |  |  |
| **78** | Brown 2011 |  |  |  |  |  |  |  | X |  |  |  |
| **79** | Hoek 2013 |  |  |  |  |  |  |  | X |  |  |  |
| **80** | Johnson 2003 |  |  |  |  |  |  |  | X |  |  |  |
| **81** | Kishchuk 2004 |  |  |  |  |  |  |  | X |  |  |  |
| **82** | Lennon 2005 |  |  |  |  |  |  |  | X |  |  |  |
| **83** | MacFadyen 2003 |  |  |  |  |  |  |  | X |  |  |  |
| **84** | Scheffels 2009 |  |  |  |  |  |  |  | X |  |  |  |
| **85** | Scheffels 2007a |  |  |  |  |  |  |  | X |  |  |  |
| **86** | Lewis 2013 |  |  |  |  |  |  |  |  | X |  |  |
| **87** | Sussman 1998 |  |  |  |  |  |  |  |  |  | X |  |
| **88** | Sussman (unpublished data) |  |  |  |  |  |  |  |  |  | X |  |
| **89** | Laoye 1972 |  |  |  |  |  |  |  |  |  | X |  |
| **90** | Skinner 1985 |  |  |  |  |  |  |  |  |  | X |  |
| **91** | Stein 1996 |  |  |  |  |  |  |  |  |  | X |  |
| **92** | Chassin 1984b |  |  |  |  |  |  |  |  |  | X |  |
| **93** | Chassin 1991c |  |  |  |  |  |  |  |  |  | X |  |
| **94** | Alexander 1983 |  |  |  |  |  |  |  |  |  | X |  |
| **95** | Ary 1988 |  |  |  |  |  |  |  |  |  | X |  |
| **96** | Green 1979 |  |  |  |  |  |  |  |  |  | X |  |
| **97** | Sargent 1998 |  |  |  |  |  |  |  |  |  | X |  |
| **98** | Zhu 1999 |  |  |  |  |  |  |  |  |  | X |  |
| **99** | Hansen 2001 |  |  |  |  |  |  |  |  |  | X |  |
| **100** | Sim 2020 |  |  |  |  |  |  |  |  |  |  | X |
| **101** | Plano Clark 2002 |  |  |  |  |  |  |  |  |  |  | X |
| **102** | Fry 2008 |  |  |  |  |  |  |  |  |  |  | X |
| **103** | Buswell 2013 |  |  |  |  |  |  |  |  |  |  | X |
| **104** | Dalum 2008 |  |  |  |  |  |  |  |  |  |  | X |
| **105** | Balch 2004 |  |  |  |  |  |  |  |  |  |  | X |
| **106** | Markham 2001 |  |  |  |  |  |  |  |  |  |  | X |
| **107** | Hong 2015 |  |  |  |  |  |  |  |  |  |  | X |
| **108** | Bhat 2018 |  |  |  |  |  |  |  |  |  |  | X |
| **109** | Seguire 2000 |  |  |  |  |  |  |  |  |  |  | X |
| **110** | Molyneux 2006 |  |  |  |  |  |  |  |  |  |  | X |
| **111** | Chang 2014 |  |  |  |  |  |  |  |  |  |  | X |
| **112** | Falkin 2007 |  |  |  |  |  |  |  |  |  |  | X |
| **113** | Highet 2004 |  |  |  |  |  |  |  |  |  |  | X |
| **114** | Vukovic 2003 |  |  |  |  |  |  |  |  |  |  | X |
| **115** | DiNapoli 2004 |  |  |  |  |  |  |  |  |  |  | X |
| **116** | Glasser 2020 |  |  |  |  |  |  |  |  |  |  | X |
| **117** | Abdullah 2006 |  |  |  |  |  |  |  |  |  |  | X |
| **118** | Morris 2011 |  |  |  |  |  |  |  |  |  |  | X |
| **119** | Patten 2009 |  |  |  |  |  |  |  |  |  |  | X |
| **120** | MacDonald 2007 |  |  |  |  |  |  |  |  |  |  | X |
| **121** | Tohid 2011 |  |  |  |  |  |  |  |  |  |  | X |
| **122** | Kulwicki 2003 |  |  |  |  |  |  |  |  |  |  | X |
| **123** | Remafedi 2005 |  |  |  |  |  |  |  |  |  |  | X |
| **124** | Denham 2004 |  |  |  |  |  |  |  |  |  |  | X |
| **125** | Panday 2003 |  |  |  |  |  |  |  |  |  |  | X |
| **126** | Peters 2011 |  |  |  |  |  |  |  |  |  |  | X |
| **127** | McVea 2009 |  |  |  |  |  |  |  |  |  |  | X |
| **128** | Seguire 2000 |  |  |  |  |  |  |  |  |  |  | X |
| **129** | Rothwell 2011 |  |  |  |  |  |  |  |  |  |  | X |
| **130** | Cosh 2015 |  |  |  |  |  |  |  |  |  |  | X |
| **131** | Constantine 2014 |  |  |  |  |  |  |  |  |  |  | X |
| **132** | Johnson 2004 |  |  |  |  |  |  |  |  |  |  | X |
| **133** | Peters 2006 |  |  |  |  |  |  |  |  |  |  | X |
| **134** | Tohid 2012 |  |  |  |  |  |  |  |  |  |  | X |
| **135** | Hauck 2013 |  |  |  |  |  |  |  |  |  |  | X |
| **136** | Grimshaw 2003 |  |  |  |  |  |  |  |  |  |  | X |
| **137** | Camenga 2015 |  |  |  |  |  |  |  |  |  |  | X |
| **138** | Antin 2019 |  |  |  |  |  |  |  |  |  |  | X |

% of overlap = 7/138 = 0.05

CA = (34+9+18+11+4+1+2+10+1+17+39)/(11x138) = 146/1518 = 0.10

CCA = (146-138)/(1518-138) = 8/1380 = 0.01
